# Innate Antiviral Immune Responses to Hepatitis B Virus

**DOI:** 10.3390/v2071394

**Published:** 2010-07-05

**Authors:** Malika Ait-goughoulte, Julie Lucifora, Fabien Zoulim, David Durantel

**Affiliations:** 1 INSERM, U871, Molecular Physiopathology and New Treatment of Viral Hepatitis, 151 Cours Albert Thomas, 69003 Lyon, France; E-Mails: malika.aitgoughoulte@inserm.fr (M.A.-g.); lucifora@virologie.med.tum.de (J.L.); fabien.zoulim@inserm.fr (F.Z.); 2 Université de Lyon, UCBL, and IFR62 Lyon Est, 69008 Lyon, France; 3 Hospices Civils de Lyon (HCL), Hôtel Dieu Hospital, 69002 Lyon, France

**Keywords:** Hepatitis B virus, innate immunity, cytokines, pathogenesis

## Abstract

Hepatitis B virus (HBV) is a major cause of acute and chronic hepatitis in humans. As HBV itself is currently viewed as a non-cytopathic virus, the liver pathology associated with hepatitis B is mainly thought to be due to immune responses directed against HBV antigens. The outcome of HBV infection is the result of complex interactions between replicating HBV and the immune system. While the role of the adaptive immune response in the resolution of HBV infection is well understood, the contribution of innate immune mechanisms remains to be clearly defined. The innate immune system represents the first line of defense against viral infection, but its role has been difficult to analyze in humans due to late diagnosis of HBV infection. In this review, we discuss recent advances in the field of innate immunity to HBV infection.

## 1. Introduction

**Figure 1:**
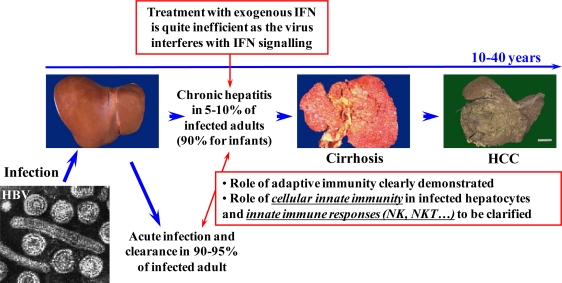
Natural history of HBV infections. HCC, hepatocellular carcinoma; NK, natural killer cells; NKT, natural killer T cells.

The outcome of hepatitis B virus (HBV) infection, as well as the severity of HBV-induced liver disease, varies widely between patients. In approximately 95 % of adults, exposure to HBV leads to an acute infection that is rapidly resolved without long-term consequences, whereas the remaining 5% fail to control viral infection, leading to chronicity. The rate of chronicity of viral infection is dramatically higher in neonates born from infected mothers, suggesting that mature immunity is important to clear infection. Patients with chronic hepatitis B (CHB) are at increased risk of developing severe liver disease, including cirrhosis and hepatocellular carcinoma (Figure 1) [1, 2]. As HBV is currently viewed as a non-cytopathic virus, HBV-associated liver damage is thought to be the consequence of a long lasting cytolytic immune response against infected hepatocytes [3, 4].

Both innate and adaptive arms of the immune system are generally involved in responding to viral infection, with innate responses being important for control of viral replication and dissemination very early after infection, as well as for timely orchestration of virus-specific adaptive responses [5]. In the case of HBV, it has been clearly shown that the adaptive response is needed for efficient and persistent control of infection [3,4]. However, the role of innate immunity has been more difficult to analyze, as HBV infection is usually diagnosed several weeks after the onset of infection when viremia is already high; thus the role of innate immunity in defense against HBV remains controversial.

The liver is composed of parenchymal cells, hepatocytes (approximately 80% of liver cells), and non-parenchymal cells (NPC), which comprise (in order of decreasing abundance) liver sinusoidal endothelial cells (LSEC), intrahepatic lymphocytes (including natural killer (NK) and natural killer T (NKT) cells), Kupffer cells (KC), biliary cells, hepatic stellate cells (HSCs), and resident dendritic cells (DCs). Due to the large number of immune cells present, the liver may be considered an immunological organ, with particular innate immune features, and is therefore thought to play an active role in the first line host defense against pathogens [6, 7]. After sensing the presence of a virus, professional innate immune cells (*i.e.*, KC, DCs, NK, NKT) produce cytokines and chemokines that have antiviral properties (e.g., IFN-α, IFN-ß, IFN-λ, TNF-α…) or that are meant to attract and stimulate adaptive immune cells (e.g., IL-2, IL-6, IL-10…). Non-professional cells (*i.e.*, LSECs, HSCs, hepatocytes) can also have immunoregulatory functions by secreting cytokines or chemokines in response to infection [8]. In general, infected cells can detect the presence of viral components or PAMPs (pathogen-associated molecular patterns) via cellular sensors or PRRs (pattern recognition receptors, such as Toll-like receptors [TLRs], RIG-like helicases [RLHs], or Nod-like receptors [NLRs]) [9,10], and produce antiviral type-I interferons (IFN) IFN-α and IFN-ß, as well as other pro-inflammatory cytokines (e.g., IL-1ß, IL-6…) [11–13]. TLRs recognize microbes either at the cell surface or on lysosome/endosome membranes, while pathogens that invade the cytosol are detected by cytoplasmic PRRs such as RLHs or NLRs [9,10]. Various TLRs are expressed in parenchymal and non-parenchymal cells of the liver [14]. Hepatocytes express mRNA for all TLRs [15,16], whereas KCs and HSCs express TLR4 and TLR2 [17,18]. In the case of lymphocytes, T and NK cells express TLR1, 2, 4, 5 and 9, whereas B cells express high levels of TLR1, 6, 7, 9 and 10 [19]. Dendritic cells can be of myeloid (mDC) or lymphoid (plasmacytoid, or pDC) origin, and represent an important component of innate immunity in the liver. Both recognize and present antigen to T cells but are distinct in their TLR expression and cytokine production profiles [19]. Plasmacytoid DCs express TLR7 and 9 and produce large amounts of IFN-α, whereas mDCs express TLR2, 3, 4 as well as 9 and produce pro-inflammatory cytokines and IFN-β but not IFN-α [19,20]. While virtually all liver cells types express RLRs [21], the pattern of expression of NLRs in hepatocytes is not known.

**Figure 2:**
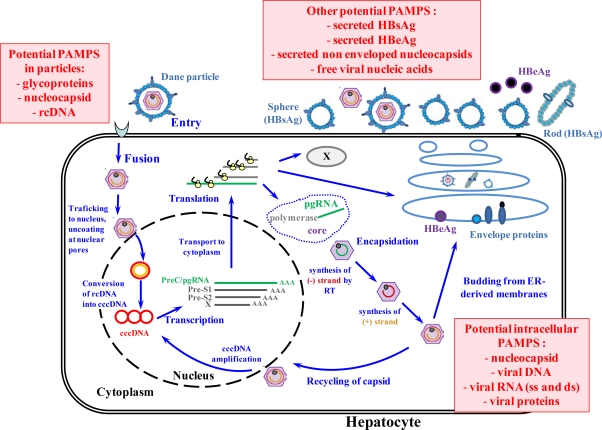
HBV life cycle and putative ‘pathogen associated molecular patterns’ (PAMPs).

Hepatitis B virus components that are sensed by hepatocytes and other liver cells are still unknown, but the putative PAMPs are highlighted in Figure 2.

The direct antiviral effect of type-I IFNs is exerted by a variety of effectors expressed from genes whose transcription is directly stimulated by IFNs, (*i.e.*, IFN-stimulated genes (ISGs)) [11–13]. The indirect antiviral effect of type-I IFNs is due to their stimulatory effect on innate and adaptive immune cells [8]. HBV is currently viewed as a stealth virus that can establish itself efficiently by evading the innate arm of the immune system. However, it remains unclear whether HBV elicits an innate response in infected hepatocytes, while escaping professional innate immune cells. In this review we will reconsider the stealth virus concept in the context of contrary findings which suggest that an innate immune response is triggered and counteracted by HBV.

## 2. HBV, a stealth virus that does not elicit innate immunity?

A characteristic feature of acute HBV infection is a prolonged incubation period during which no apparent clinical symptoms or biochemical manifestations of liver injury are observed. Indeed HBV-infected patients are almost universally diagnosed after the onset of clinical symptoms, which occur 10 to 12 weeks after infection [22]. Studies performed with animal models, including the woodchuck model [23] and HBV-infected chimpanzees [24], have suggested that viral replication remains largely undetectable until 3–4 weeks post-infection, then “explodes” infecting almost all hepatocytes. These studies, together with studies performed with patient samples [3,4], clearly establish the role of adaptive immunity for clearance of the virus after acute infection. In contrast, the potential role of innate responses has been difficult to analyze, because of the difficulty of finding a cohort of patients in the acute phase. The reasons for the delayed appearance of measurable levels of HBV proteins and DNA in the first weeks of infection are not clear. It has been suggested that immediately after infection, HBV could be retained in other organs before reaching the liver, as described for the woodchuck hepatitis virus (WHV), for which the initial site of infection has been reported to be the bone marrow [25]. However this possibility is still speculative as the lymphotropism of WHV is more pronounced than that of HBV [25,26]. Another possibility is that HBV might initially infect very few hepatocytes, then spread very slowly throughout the liver. This lag phase, without measurable viremia, is difficult to study during human HBV infection.

Using experimentally infected chimpanzees, microarray analyses have suggested that HBV, early in infection, does not modify host cellular gene transcription and does not induce innate antiviral responses in hepatocytes and the liver [27]. In this study, global gene expression profiling was performed using liver RNA obtained at multiple time points after infection of three chimpanzees. All three infected chimpanzees developed a self-limited infection, reaching very similar viral titers and cleared the virus with relatively similar kinetics profiles. The clearance of the virus was clearly associated with an efficient adaptive immune response. After establishing the gene expression profiles for all animals, genes with expression patterns correlated with the amount of HBV DNA in the liver of all three animals over the entire time course could not be identified. The failure of the virus to induce cellular gene expression as it spread through the liver suggested that HBV behaves as a stealth virus, capable of evading the first line of host defences [24]. HBV infection clearly contrasted with hepatitis C virus (HCV) infection in the same model; indeed, HCV infection is accompanied by a profound modification of cellular gene expression, in particular of ISGs. It was proposed that the invisibility of HBV to the innate sensing machinery of the cells could be partially attributable to its replication strategy. First of all, templates for HBV transcription are retained in the nucleus. Second, transcription of viral genes involves the production of capped and polyadenylated viral mRNAs that resemble the structure of normal cellular transcripts. And third, HBV sequesters its genome within viral capsid particles in the cytoplasm [28]. However, a role for the innate immune response in the control of early HBV replication should not be dismissed as the expression of the relevant genes might occur below the level of detection of the microarray analysis and/or in a limited number of cells at a given time point. Whether these observations can also be translated to the pathogenesis of natural human infection is still unknown. HBV hepatitis is generally milder in chimpanzees than in humans, and it is possible that the inability to detect activation of genes related to innate immunity reflects the milder disease manifestation.

So far no gene expression analysis has been performed in the human setting during the acute phase. However, a few studies have quantified circulating innate cytokines/chemokines and analysed the function of circulating NK/NKT cells in patients with acute HBV infection in order to measure the early kinetics of innate immunity. In a study reported by Stacey and collaborators, the immune response induced during the initial stages of infection was characterised by performing kinetic quantification of circulating innate cytokines/chemokines on plasma samples of 35 HIV, 10 HBV, as well as 10 HCV patients in the acute phase of infection [29]. This work showed striking differences in the pattern of elevation in cytokine and chemokine levels observed in plasma during the phase of exponential viral amplification. Indeed, a strong and rapid induction of classic innate cytokines followed by multiple other cytokines was detected in acute HIV infection whereas weaker perturbation in plasma cytokine levels was observed in acute HBV infection. Cytokine levels after HCV infection were delayed and less intense compared to that observed in acute HIV infection, but more intense than that observed for HBV. Although weaker, the production of cytokines/chemokines after HBV infection was not null. Several HBV patients produced detectable levels of systemic IFN-α, TNF-α, IL-15, IL-10, IL-6, and/or IL-1β within 10 days after initiation of viral expansion and before the peak of viremia, suggesting that a significant innate response to HBV can be detected in some patients. In contrast, another recent study, performed on 21 HBV patients during the pre-symptomatic phase, showed that type-I IFNs, IL-15, and IFN-λ1 were not appropriately induced before or concomitantly with the peak of viremia, as compared to the systemic induction observed during hepatitis A virus (HAV) infection [30]. Interestingly, the level of serum IFN/IL-15 was found to be lower at peak viremia than during the resolution of disease, suggesting that HBV may be able to inhibit the production of these cytokines. In these same patients, The peak of viremia coincided with high levels of IL-10, an anti-inflammatory cytokine involved in the inhibition of NK and T-cell functions. Altogether the authors of this study concluded that the virus was not able to elicit a strong production IFN/IL-15 cytokines, but did induce the production of IL-10. They proposed that, in addition to failing to induce some immune mechanisms, HBV uses an immunosuppressive strategy to actively inhibit others [30].

The results obtained in these HBV studies deserve some discussion. Since HBV replicates in the liver, in contrast to HIV which replicates mainly in PBMC, it is not surprising that cytokines produced by innate immune cells are not found at the systemic level. That HAV, another hepatotropic virus, does induce a systemic production of type-I IFN is not really informative, as HAV is not capable of inducing chronic infection and may not have evolved successful strategies to counteract host immunity. Based on published literature, it seems that HBV induces a cytokine response that is more pronounced in humans than in chimpanzees. The production of cytokines in response to infection, including IL-10, suggests that HBV can be detected in some way by the host. However, it will be difficult to address these questions in more detail, as the study of gene expression in serial liver biopsy specimens of patients experiencing an acute HBV infection will not be ethically feasible.

As a characteristic of chronic infection, HBV appears to be able to induce a long-lasting inhibition of innate immunity. A recent clinical study investigated the immune mechanisms acting during the pathogenesis of spontaneous or antiviral withdrawal-induced hepatic flares (*i.e.*, sudden changes of HBV viremia) in chronically infected patients. Signs of immune reactivation were almost completely absent during the rebound of HBV replication [31]. Serum levels of pro-inflammatory cytokines (IL-1, TNF-α, IL-6 and IFN-α) were measured and were consistently normal. Only IFN-γ inducible chemokines CXCL-9 and -10 were found increased in the serum of patients experiencing these hepatic flares. Although this study was performed in patients undergoing a reactivation of HBV replication (with titers increased from 10^2–3^ to 10^9–10^) and not during primary infection, the findings are consistent with the idea that HBV might escape innate immune recognition. However, it is worth noting that no quantification of HBsAg or HBeAg was reported during the observed flares. Since these molecules have putative immunomodulatory functions, including the inhibition of innate immune cells, it would have been interesting to follow their serum levels to probe for possible mechanisms of immune evasion.

To summarize, HBV, in contrast to HCV, does not seem to extensively modify gene expression in the liver during the acute phase and does not elicit a strong innate immune response, as evidenced by the low level of innate immune cytokines detected systemically (*i.e.*, in serum). However, one cannot exclude the possibility that HBV induces a modest local modification of gene expression in immune cells or hepatocytes leading to the local production of innate immune cytokines, including cytokines with antiviral properties.

## 3. Can HBV, as other viruses, be sensed by the immune system?

A recent study performed in the woodchuck model showed that woodchuck hepatitis virus (WHV) could be detected by the innate immune system. Both natural killer and natural killer T cell responses could be mounted soon after infection (*i.e.*, hours p.i.) with a high dose of virus [32]. These responses were at least partially capable of limiting viral propagation (*i.e.*, causing a transient reduction of viremia), but were not followed by a prompt adaptive T-cell response, which occurred with a delay of 4 to 5 weeks. These results suggest that, despite a potential very early detection of the infection by the innate immune system, WHV would be able to induce immune tolerance and delay the adaptive response. Therefore, rather than being silent, hepadnaviruses could very efficient counteract the innate immune response, thus preventing the secretion of cytokines during the early phases of infection. Somehow weakening these observations, it is worth noting that, in this study, high doses of virus were used for the inoculation of woodchucks (*i.e.*, 1.1×10^10^), which clearly exceeds a physiological infectious dose. The two human studies described above showed i) that one patient had elevated levels of IL-15 and NK cell activation and function just before or at the peak viremia [30] and ii) that in approximately 50% of subjects a detectable level of systemic IFN-α, TNF-α, IL-15, IL-10, IL-6, and IL-1β could be detected within 10 days after initiation of viral expansion and before the peak of viremia [29], suggesting that the virus could be sensed by the innate immune system in those HBV infected patients.

Mice transgenic for replication-competent HBV genome expressed in their hepatocytes have been used to study the antiviral effects of type I interferon [33]. Indeed, IFN-α and IFN-ß-induced responses, in this model, inhibited the formation of new HBV capsids, destabilized existing capsids, and degraded preformed HBV RNA [33]. Further indirect observations have also suggested that innate immunity could be important in the natural control of HBV replication. In HBV-transgenic mice deficient for IFNAR1, PKR or IRF1, which are components of the innate response, HBV replication was highly increased as compared to that observed in wild type HBV transgenic mice [34]. Recent genetic studies performed in humans have also shown that polymorphisms in the *ifnar1* gene correlated with an increased susceptibility to chronic hepatitis B (CHB) [35,36].

*In vitro*, two main systems can be used to study HBV replication: primary human hepatocytes (PHH) [37] and cell lines of liver progenitor (*i.e.*, HepaRG [38]). Using isolated PHH (with low level of contamination with non parenchymal cells, NPC) and NPC, Hösel *et al.* investigated whether and how HBV was detected by parenchymal and/or non parenchymal cells and analyzed downstream events [39]. It was shown that HBV was recognized by KC, although the virus does not replicate in these cells, and that within hours post infection, this recognition leads to the activation of NFκB and subsequently to the release of IL-6 and other pro-inflammatory cytokines (*i.e.*, IL-8, TNFα, IL-1ß). Interestingly, in this experimental setting, no induction of type-I interferon (*i.e.*, IFN-β) was observed. The activation of IL-6 and other pro-inflammatory cytokines was transient and inhibited responsiveness to a subsequent challenge. The IL-6 released by KC after the activation of NFκB was shown to control HBV gene transcription and replication in hepatocytes shortly after infection. Mechanistic analysis revealed that IL-6 activated the mitogen-activated protein kinases ERK1/2 and JNK, which in turn inhibited the expression of hepatocyte nuclear factor (HNF) 1α and HNF 4α, two transcription factors essential for HBV gene expression and replication [39]. It was suggested that IL-6 could ensure an early control of virus replication, thereby limiting the activation of the adaptive immune response and preventing death of the HBV-infected hepatocytes in the early phase of infection. This hypothesis fits well with the already described protective effect of IL-6 in the context of liver failure [40]. Alternatively, the production of IL-6 could be the hallmark of a tentative attempt by the host to inhibit HBV replication and clear viral infection. Interestingly, the production of IL-6 and other cytokines seems transient after HBV infection, and HBV replication tends to increase after 3–4 days post infection when IL-6 level has already returned to baseline. This suggests that the virus actively counteracts the effects of IL-6. Hence, like the human cytomegalovirus [41], HBV may have evolved mechanisms to modulate the expression or signaling of IL-6 as part of the viral arsenal of immune evasion strategies.

It is somewhat surprising that HBV does not seem to induce the production of type-I IFN in infected primary human liver cells, as this cytokine is frequently produced and secreted by cells infected by viruses [11–13]. Interestingly, during the initial phase after viral entry, there appears to be a temporary block of HBV replication and spread [42]. It remains possible that this is partially mediated by innate immune mechanisms. Hösel *et al.* have obtained results suggesting that the transcription of the IFN-β gene is not induced by HBV infection in PHH and/or KCs [39]. It is worth noting that in PHH, as well as in HepaRG cells, the overall replication level is rather low, with approximately 20% of cells infected (as detected by immunostaining), which complicates the study of the host/pathogen interaction [37,38]. One could hypothesize that the low level of replication might be the consequence of an innate cellular response. In this case, the virus would trigger a host antiviral response that would limit HBV replication to only a low percentage of cells. This low percentage of infected cells is an obstacle for studying the potential ability of HBV to elicit a type-I IFN response. Indeed, in other viral models, when a low multiplicity of infection is used, which is likely the case during natural infection by HBV, it has been documented that an IFN response may occur in only a low percentage (<30%) of infected cells [43]. Moreover, some viruses are particularly efficient at counteracting this IFN response and may therefore render the analysis of IFN response more difficult [44]. This could be the case for HBV which has been shown to be very efficient at inhibiting the IFN signalling pathway [45–47]. Another technical obstacle for studying the potential ability of HBV to elicit an IFN response is that an inoculation time of 12–16 hours is required to initiate a strong infection of cultured hepatocytes *in vitro*. This is not compatible with the very early post-infection and synchronized analysis that would likely be necessary to detect a potentially weak and transient IFN response. To test the hypothesis that HBV can elicit and then disarm a type-I IFN response in cell culture, it seemed necessary to initiate time-controlled and high-level HBV replication in a large number of cells. Hence, Lucifora *et al.*, performed a study in HepaRG cells replicating HBV at high level after transduction of the cells by a recombinant baculovirus carrying the whole HBV genome (*i.e.*, Bac-HBV) [48]. It was shown that hepatocytes, in the absence of NPC, can mount an innate antiviral response which results in a non-cytopathic clearance of HBV DNA [49]. Cellular gene expression analyses showed that IFN-ß and other interferon stimulated genes were up-regulated in HepaRG cells transduced with Bac-HBV, but not in cells transduced by control baculovirus. Confirming the role of IFN-ß, viral replication was rescued when IFN-ß action was inhibited either by neutralizing antibodies or RNA interference targeting the type-I IFN receptor. This baculoviral expression system showed that HBV replication could elicit a type-I IFN response in infected cells. This model utilizes overexpression of HBV, so the results need to be confirmed under more physiological conditions. The demonstration of very early virus-host cell interactions has been hampered by the lack of a powerful infection system based on physiologically relevant cells.

A very recent study described innate and adaptive human responses *in vivo* in the incubation phase of HBV [50]. This study, performed on two seronegative blood donors who became HBsAg and HBV DNA positive without elevation of ALT and who were monitored at very early stage of infection, clearly established that the human innate immune system is also able to sense HBV infection and develop NK and NKT cell responses [50]. These observations are consistent with a previous longitudinal analysis of circulating NK cells showing that their frequency is elevated in the incubation period of natural HBV infection with a subsequent decline at the time of the decrease in HBV DNA [22].

**Figure 3:**
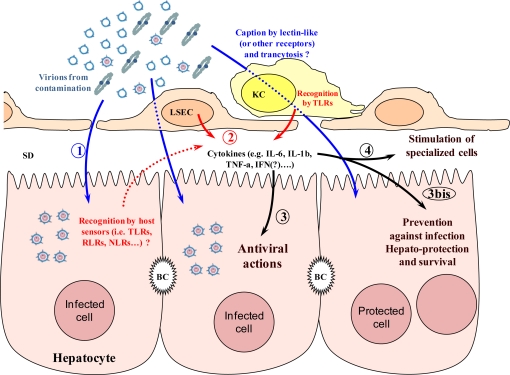
**Overview of early events after HBV infection.** 1. Infection step (direct or via liver sinusoidal endothelial (LSEC) or Kupffer (KC) cells taking up and presentation). 2. Recognition step by toll like (TLRs), RIG-like (RLRs) or Nod-like (NLRs) receptors. 3. Direct antiviral action of produced cytokines. 3bis. Cell protection against infection. 4. Cross talk with specialized cells. BC, Bile caniculus; SD, Space of Dissé.

Altogether, data reported with animal models or *in vitro* suggest that HBV can be sensed by the immune system early in infection and this response could be important for controlling HBV replication (Figure 3). The recently observed induction of cytokine and NK/NKT cell responses in humans suggests that, rather than being silent, HBV would be able to induce and counteract the action of the immune system.

## 4. Can HBV inhibit type-I interferon pathways?

Only approximately one third of patients with CHB respond to IFN-α treatment. The cause of treatment failure in non-responders is not fully understood, but the interference of HBV with IFN-α-induced JAK–STAT signaling has emerged as a possible escape strategy of HBV contributing to viral persistence and disease progression [45]. Several studies have reported HBV interference with IFN signaling pathways *in vitro*, using overexpression and/or non replicating systems, providing interesting insights about the molecular mechanisms used by HBV to counteract the effects of IFN. Most of these data, however, need to be confirmed *in vivo*. The first experimental evidence that HBV can interfere with IFN pathways came in the late 80’s to early 90’s. Twu and colleagues reported that HBV was able to inhibit the transcription of the *ifn-β* gene in hepatoma cells [51], and identified the core protein as the main viral determinant of this inhibition [52]. Later, other groups also highlighted the involvement of HBV core protein in the inhibition of the IFN pathway. It was shown that the MxA protein, an effector of the IFN response, is totally inhibited in the HBV-replicating HepG2.2.15 cell line [46,53], in peripheral blood mononuclear cells (PBMC) from HBV chronically infected patients [54], as well as in HepG2 cells transfected with a re-circularized HBV DNA [46]. Moreover, co-transfection of an MxA promoter-driven reporter construct with an HBV core protein construct demonstrated, upon IFN induction, a clear inhibition of MxA expression [46]. This inhibition was due to a direct binding of the core protein with the ISRE sequence of the human MxA promoter [46]. Similar results were obtained in cells transfected with defective HBV genome. Defective HBV particles originating from the encapsidation of spliced HBV RNA are detected in the serum of hepatitis patients, and more abundantly in patients with acute hepatitis progressing to CHB or with established CHB, as compared to recovering patients [55,56]. This suggests a potential role for the establishment of chronicity. *In vitro*, it was demonstrated that the expression of HBV DNA leads to a cytoplasmic accumulation of the core protein which strongly reduces the antiviral activity of IFN-α via the inhibition of MxA expression [47].

**Figure 4:**
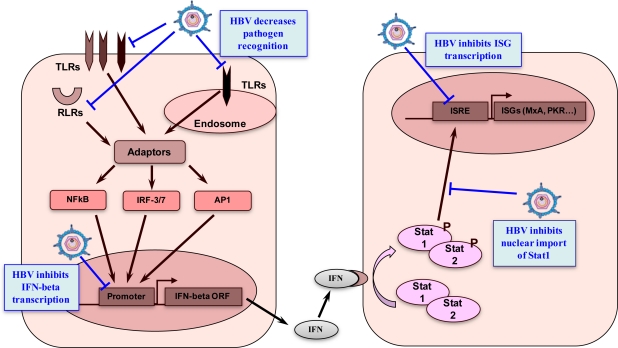
Inhibition of host sensors and IFN pathway by HBV.

Beside core, other HBV proteins have been implicated in the inhibition of IFN pathway. For instance, it was shown that cells transfected by the terminal protein domain of the HBV viral polymerase are resistant to IFN-α, IFN-γ and dsRNA stimulation [57]. Furthermore, the expression of the terminal protein in the liver of infected patients is associated with a failure of hepatocytes to respond to interferon [58]. It is worth noting, however, that later *in vitro* experiments failed to reproduce these observations, thus further investigation is warranted [59]. However, a recent study demonstrates that the HBV polymerase, and especially the terminal protein domain, inhibits the expression of the IFN-α-inducible protein MyD88 by interfering with target promoter activity through blockage of Stat1 nuclear import. Moreover, transcriptional activity of the ISRE promoter and expression of ISGs such as STAT1 and ISG15 were also inhibited [60]. HBV S and/or X proteins also seem to be involved in the inhibition of the IFN pathway. Indeed, using a cell line that allows the controlled expression of HBV and liver biopsies from patients chronically infected by HBV, it has been shown that HBV, and in particular S and/or X proteins, could also block STAT1 nuclear import by up-regulation of a cellular protein named pp2AC [45]. Similar results were previously reported by the same group using liver extracts from HCV transgenic mice and patients with CHC [61]. It has also been demonstrated that HBs antigens may affect the function of monocytes through inhibition of the NFκB and ERK pathway [62]. Altogether, these data demonstrate that various HBV proteins can counteract the innate immune response by inhibiting different components of the IFN signaling pathway (Figure 4). As most of these data were generated *in vitro* and not in the context of a proper infection, the relevance of these observations still needs to be analyzed in a more physiologic setting.

## 5. Can HBV inhibit host sensors of the innate response?

Beside the inhibition of the downstream IFN signaling pathways, there is a growing body of evidence suggesting that HBV can also interfere with the host recognition systems (Figure 4). Upon recognition of PAMPs, and as mentioned before, PRRs induce the production of IFN and other cytokines of the innate immune response and are therefore major players in the innate response [63]. Recently, interesting data have been generated showing that HBV can modulate the expression of TLRs and/or inhibit TLR signaling cascades, suggesting that HBV might use this strategy to escape the innate immune response. A downregulation of TLR-2 expression in PBMCs, as well as in KCs and hepatocytes from HBeAg positive patients was recently described [64]. This downregulation was associated with HBeAg positivity, which suggests an immunomodulatory role for this secreted viral protein. Interestingly in the HBV transgenic mouse model the only TLR ligand that did not induce an anti-HBV effect was the TLR-2 ligand [65]. This result could be explained by the HBV-induced downregulation of this receptor. This finding was confirmed and extended by recent data showing that PBMCs from patients with CHB expressed significantly lower levels of TLR-1, TLR-2, TLR-4 and TLR-6 mRNA compared to those from healthy donors [66]. Moreover the production of cytokines in response to both TLR2 and TLR4 ligands by PBMCs from CHB patients was inhibited and there was a correlation between this inhibition and the level of HBsAg [66]. Finally, using the HBV transgenic mouse model, it was also demonstrated that HBV (either purified virions, HBeAg or HBsAg) suppresses the innate response elicited by TLR3 and TLR4 stimulation of hepatocytes and non parenchymal liver cells [67]. This correlated with the suppression of IFN-β production and subsequent decrease in the activation of interferon-stimulated genes (e.g., MxA, IP-10, NFκB, or pERK) [67].

Amongst PBMCs, pDCs are the major type-I interferon producing cells and a key sensor of viral infections as they express both TLR7 and TLR9, TLRs that recognise, even in absence of viral replication, ssRNA and unmethylated CpG motifs, respectively [68]. A recent study has reported a reduction of TLR9 expression in pDCs of patients with CHB, which correlated with impaired IFN-α production [69]. Another study, this one using monocyte-derived dendritic cells (MoDCs) from CHB, analyzed the involvement of TLR3 in HBV infection and demonstrated that TLR3 was downregulated markedly in MoDCs from these patients [70]. Furthermore, the production of IFN-ß, a downstream cytokine of TLR3 signalling, was also reduced in MoDCs from CHB patients [70]. Altogether, these data suggest that HBV could alter the innate immune response triggered by both specialized cells and hepatocytes by inhibiting TLR functions, which may contribute to the establishment of chronic infections. However, other studies reported minor alterations of DC functions during CHB [71,72]. HBV-specific CD4^+^ and CD8^+^ T cells become detectable a short time after the exponential increase in HBV replication [22], suggesting that DC function is not affected by the virus at early phases of self-limited HBV infection. These discrepancies might reflect differences between experimental model systems, particularly in the case of the generation of DC from monocytic precursors. During preparation, artifacts may occur resulting in a cell population containing DC and non-DC; therefore, these studies have to be interpreted with caution. Also, whether these data are applicable to DC subpopulations *in vivo* still needs to be determined.

Altogether, these data demonstrate that HBV proteins can counteract the innate immune response by inhibiting sensors, mediators, as well as effectors of the cellular antiviral innate response *in vitro.* One may hypothesize that HBV has evolved these mechanisms in its encounters with this type of response during its viral cycle *in vivo*.

## 6. Concluding remarks

In most immunocompetent adults, an acute HBV infection is resolved by an efficient adaptive immune response. Usually innate immune responses are necessary to mount a rapid and efficient adaptive immune response. In the case of HBV, the study of the role of the innate immunity has been complicated by the fact that patients are usually diagnosed long after the onset of infection.

It is difficult to imagine that HBV would not elicit any innate immune response at all. However, HBV is generally believed to be a stealth virus that does not modulate host cellular gene transcription and does not induce an innate immune response when spreading throughout the liver. The available data suggest three possibilities: i) HBV originally evolved to evade innate immunity by not inducing it; ii) an innate immune response is elicited by HBV, as by other viruses, but at a lower extent and is therefore difficult to detect at the level of the liver sinusoid (local events); or iii) HBV induces an innate immune response but is rapidly capable to inhibit it. Such early events are difficult to analyze during natural infection in humans. However, recent *in vitro* and *in vivo* observations provide new pieces of evidence suggesting that HBV could be sensed by the immune system in humans during early infection and in animal models. The liver cells, including non-parenchymal cells and hepatocytes, might be able to sense HBV infection and mount an antiviral response, notably via an interferon response. The sensitivity of HBV replication to interferon has clearly been demonstrated *in vitro* and *in vivo*. This immune response may lead to epigenetic regulation of the transcriptional activity of the HBV covalently-closed-circular DNA or to other transcriptional or translational effects leading to the downregulation of viral gene expression and replication. Alternatively, in order to establish a chronic infection, HBV may evade this innate response or downregulate antiviral pathways, such as the interferon pathway, thus explaining some of the apparently contradictory results published in the literature. The experimental conditions, timing, and types of analyses are major variables that may have contributed to the controversy. Since all these findings may have major clinical implications for the immunological control of viral infection, more studies are warranted in relevant study models to better characterize the innate sensors detecting HBV, the innate effectors induced by HBV, the viral determinants inducing the innate response, and the mechanisms employed by HBV to counteract cellular responses in order to establish persistent infection and resistance to exogenous IFNα administration.

## References

[1] Dienstag JL (2008). Hepatitis B virus infection. N Engl J Med.

[2] Lavanchy D (2005). Worldwide epidemiology of HBV infection, disease burden, and vaccine prevention. J Clin Virol.

[3] Bertoletti A, Gehring AJ (2006). The immune response during hepatitis B virus infection. J Gen Virol.

[4] Rehermann B, Nascimbeni M (2005). Immunology of hepatitis B virus and hepatitis C virus infection. Nat Rev Immunol.

[5] Biron CA, Sen GN, Knipe DM, Howley PM (2007). Innate responses to viral infections. Fields Virology.

[6] Gao B, Jeong WI, Tian Z (2008). Liver: An organ with predominant innate immunity. Hepatology.

[7] Racanelli V, Rehermann B (2006). The liver as an immunological organ. Hepatology.

[8] Crispe IN (2009). The liver as a lymphoid organ. Annu Rev Immunol.

[9] Akira S, Uematsu S, Takeuchi O (2006). Pathogen recognition and innate immunity. Cell.

[10] Kawai T, Akira S (2006). Innate immune recognition of viral infection. Nat Immunol.

[11] Randall RE, Goodbourn S (2008). Interferons and viruses: an interplay between induction, signalling, antiviral responses and virus countermeasures. J Gen Virol.

[12] Samuel CE (2001). Antiviral actions of interferons. Clin Microbiol Rev.

[13] Martinon F, Mayor A, Tschopp J (2009). The inflammasomes: guardians of the body. Annu Rev Immunol.

[14] Zarember KA, Godowski PJ (2002). Tissue expression of human Toll-like receptors and differential regulation of Toll-like receptor mRNAs in leukocytes in response to microbes, their products, and cytokines. J Immunol.

[15] Liu S, Gallo DJ, Green AM, Williams DL, Gong X, Shapiro RA, Gambotto AA, Humphris EL, Vodovotz Y, Billiar TR (2002). Role of toll-like receptors in changes in gene expression and NF-kappa B activation in mouse hepatocytes stimulated with lipopolysaccharide. Infect Immun.

[16] Nishimura M, Naito S (2005). Tissue-specific mRNA expression profiles of human ATP-binding cassette and solute carrier transporter superfamilies. Drug Metab Pharmacokinet.

[17] Ojaniemi M, Liljeroos M, Harju K, Sormunen R, Vuolteenaho R, Hallman M (2006). TLR-2 is upregulated and mobilized to the hepatocyte plasma membrane in the space of Disse and to the Kupffer cells TLR-4 dependently during acute endotoxemia in mice. Immunol Lett.

[18] Paik YH, Schwabe RF, Bataller R, Russo MP, Jobin C, Brenner DA (2003). Toll-like receptor 4 mediates inflammatory signaling by bacterial lipopolysaccharide in human hepatic stellate cells. Hepatology.

[19] Hornung V, Rothenfusser S, Britsch S, Krug A, Jahrsdorfer B, Giese T, Endres S, Hartmann G (2002). Quantitative expression of toll-like receptor 1–10 mRNA in cellular subsets of human peripheral blood mononuclear cells and sensitivity to CpG oligodeoxynucleotides. J Immunol.

[20] Vogel SN, Fitzgerald KA, Fenton MJ (2003). TLRs: differential adapter utilization by toll-like receptors mediates TLR-specific patterns of gene expression. Mol Interv.

[21] Iwasaki A, Medzhitov R Regulation of adaptive immunity by the innate immune system. Science.

[22] Webster GJ, Reignat S, Maini MK, Whalley SA, Ogg GS, King A, Brown D, Amlot PL, Williams R, Vergani D, Dusheiko GM, Bertoletti A (2000). Incubation phase of acute hepatitis B in man: dynamic of cellular immune mechanisms. Hepatology.

[23] Menne S, Cote PJ (2007). The woodchuck as an animal model for pathogenesis and therapy of chronic hepatitis B virus infection. World J Gastroenterol.

[24] Wieland SF, Chisari FV (2005). Stealth and cunning: hepatitis B and hepatitis C viruses. J Virol.

[25] Coffin CS, Michalak TI (1999). Persistence of infectious hepadnavirus in the offspring of woodchuck mothers recovered from viral hepatitis. J Clin Invest.

[26] Lew YY, Michalak TI (2001). *In vitro* and *in vivo* infectivity and pathogenicity of the lymphoid cell-derived woodchuck hepatitis virus. J Virol.

[27] Wieland S, Thimme R, Purcell RH, Chisari FV (2004). Genomic analysis of the host response to hepatitis B virus infection. Proc Natl Acad Sci U S A.

[28] Guidotti LG, Chisari FV (2006). Immunobiology and pathogenesis of viral hepatitis. Annu Rev Pathol.

[29] Stacey AR, Norris PJ, Qin L, Haygreen EA, Taylor E, Heitman J, Lebedeva M, DeCamp A, Li D, Grove D, Self SG, Borrow P (2009). Induction of a striking systemic cytokine cascade prior to peak viremia in acute human immunodeficiency virus type 1 infection, in contrast to more modest and delayed responses in acute hepatitis B and C virus infections. J Virol.

[30] Dunn C, Peppa D, Khanna P, Nebbia G, Jones M, Brendish N, Lascar RM, Brown D, Gilson RJ, Tedder RJ, Dusheiko GM, Jacobs M, Klenerman P, Maini MK (2009). Temporal analysis of early immune responses in patients with acute hepatitis B virus infection. Gastroenterology.

[31] Tan AT, Koh S, Goh W, Zhe HY, Gehring AJ, Lim SG, Bertoletti A A longitudinal analysis of innate and adaptive immune profile during hepatic flares in chronic hepatitis B. J Hepatol.

[32] Guy CS, Mulrooney-Cousins PM, Churchill ND, Michalak TI (2008). Intrahepatic expression of genes affiliated with innate and adaptive immune responses immediately after invasion and during acute infection with woodchuck hepadnavirus. J Virol.

[33] McClary H, Koch R, Chisari FV, Guidotti LG (2000). Relative sensitivity of hepatitis B virus and other hepatotropic viruses to the antiviral effects of cytokines. J Virol.

[34] Guidotti LG, Morris A, Mendez H, Koch R, Silverman RH, Williams BR, Chisari FV (2002). Interferon-regulated pathways that control hepatitis B virus replication in transgenic mice. J Virol.

[35] Zhou J, Lu L, Yuen MF, Lam TW, Chung CP, Lam CL, Zhang B, Wang S, Chen Y, Wu SH, Poon VK, Ng F, Chan CC, Jiang S, Yuen KY, Zheng BJ (2007). Polymorphisms of type I interferon receptor 1 promoter and their effects on chronic hepatitis B virus infection. J Hepatol.

[36] Zhou J, Smith DK, Lu L, Poon VK, Ng F, Chen DQ, Huang JD, Yuen KY, Cao KY, Zheng BJ (2009). A non-synonymous single nucleotide polymorphism in IFNAR1 affects susceptibility to chronic hepatitis B virus infection. J Viral Hepat.

[37] Gripon P, Diot C, Theze N, Fourel I, Loreal O, Brechot C, Guguen-Guillouzo C (1988). Hepatitis B virus infection of adult human hepatocytes cultured in the presence of dimethyl sulfoxide. J Virol.

[38] Gripon P, Rumin S, Urban S, Le Seyec J, Glaise D, Cannie I, Guyomard C, Lucas J, Trepo C, Guguen-Guillouzo C (2002). Infection of a human hepatoma cell line by hepatitis B virus. Proc Natl Acad Sci U S A.

[39] Hosel M, Quasdorff M, Wiegmann K, Webb D, Zedler U, Broxtermann M, Tedjokusumo R, Esser K, Arzberger S, Kirschning CJ, Langenkamp A, Falk C, Buning H, Rose-John S, Protzer U (2009). Not interferon, but interleukin-6 controls early gene expression in hepatitis B virus infection. Hepatology.

[40] Luedde T, Trautwein C (2006). Intracellular survival pathways in the liver. Liver Int.

[41] Gealy C, Denson M, Humphreys C, McSharry B, Wilkinson G, Caswell R (2005). Posttranscriptional suppression of interleukin-6 production by human cytomegalovirus. J Virol.

[42] Bertoletti A, Ferrari C (2003). Kinetics of the immune response during HBV and HCV infection. Hepatology.

[43] Apostolou E, Thanos D (2008). Virus Infection Induces NF-kappaB-dependent interchromosomal associations mediating monoallelic IFN-beta gene expression. Cell.

[44] Mossman KL, Macgregor PF, Rozmus JJ, Goryachev AB, Edwards AM, Smiley JR (2001). Herpes simplex virus triggers and then disarms a host antiviral response. J Virol.

[45] Christen V, Duong F, Bernsmeier C, Sun D, Nassal M, Heim MH (2007). Inhibition of alpha interferon signaling by hepatitis B virus. J Virol.

[46] Fernandez M, Quiroga JA, Carreno V (2003). Hepatitis B virus downregulates the human interferon-inducible MxA promoter through direct interaction of precore/core proteins. J Gen Virol.

[47] Rosmorduc O, Sirma H, Soussan P, Gordien E, Lebon P, Horisberger M, Brechot C, Kremsdorf D (1999). Inhibition of interferon-inducible MxA protein expression by hepatitis B virus capsid protein. J Gen Virol.

[48] Lucifora J, Durantel D, Belloni L, Barraud L, Villet S, Vincent IE, Margeridon-Thermet S, Hantz O, Kay A, Levrero M, Zoulim F (2008). Initiation of hepatitis B virus genome replication and production of infectious virus following delivery in HepG2 cells by novel recombinant baculovirus vector. J Gen Virol.

[49] Lucifora J, Durantel D, Testoni B, Hantz O, Levrero M, Zoulim F (2009). Control of hepatitis B virus replication by innate response of HepaRG cells. Hepatology.

[50] Fisicaro P, Valdatta C, Boni C, Massari M, Mori C, Zerbini A, Orlandini A, Sacchelli L, Missale G, Ferrari C (2009). Early kinetics of innate and adaptive immune responses during hepatitis B virus infection. Gut.

[51] Twu JS, Lee CH, Lin PM, Schloemer RH (1988). Hepatitis B virus suppresses expression of human beta-interferon. Proc Natl Acad Sci U S A.

[52] Whitten TM, Quets AT, Schloemer RH (1991). Identification of the hepatitis B virus factor that inhibits expression of the beta interferon gene. J Virol.

[53] Guan SH, Lu M, Grunewald P, Roggendorf M, Gerken G, Schlaak JF (2007). Interferon-alpha response in chronic hepatitis B-transfected HepG2.2.15 cells is partially restored by lamivudine treatment. World J Gastroenterol.

[54] Fernandez M, Quiroga JA, Martin J, Cotonat T, Pardo M, Horisberger MA, Carreno V (1997). Impaired interferon induction of human MxA protein in chronic hepatitis B virus infection. J Med Virol.

[55] Rosmorduc O, Petit MA, Pol S, Capel F, Bortolotti F, Berthelot P, Brechot C, Kremsdorf D (1995). *In vivo* and *in vitro* expression of defective hepatitis B virus particles generated by spliced hepatitis B virus RNA. Hepatology.

[56] Soussan P, Pol J, Garreau F, Schneider V, Pendeven CL, Nalpas B, Lacombe K, Bonnard P, Pol S, Kremsdorf D (2008). Expression of Defective Hepatitis B Virus Particles Derived from Singly Spliced RNA Is Related to Liver Disease. J Infect Dis.

[57] Foster GR, Ackrill AM, Goldin RD, Kerr IM, Thomas HC, Stark GR (1991). Expression of the terminal protein region of hepatitis B virus inhibits cellular responses to interferons alpha and gamma and double-stranded RNA. Proc Natl Acad Sci U S A.

[58] Foster GR, Goldin RD, Hay A, McGarvey MJ, Stark GR, Thomas HC (1993). Expression of the terminal protein of hepatitis B virus is associated with failure to respond to interferon therapy. Hepatology.

[59] Foster GR, Ackrill AM, Goldin RD, Kerr IM, Thomas HC, Stark GR (1995). Corections : expression of the terminal protein region of hepatitis B virus inhibits cellular responses to interferons alpha and gamma and double-stranded RNA. Proc Natl Acad Sci U S A.

[60] Wu M, Xu Y, Lin S, Zhang X, Xiang L, Yuan Z (2007). Hepatitis B virus polymerase inhibits the interferon-inducible MyD88 promoter by blocking nuclear translocation of Stat1. J Gen Virol.

[61] Duong FH, Filipowicz M, Tripodi M, La Monica N, Heim MH (2004). Hepatitis C virus inhibits interferon signaling through up-regulation of protein phosphatase 2A. Gastroenterology.

[62] Cheng J, Imanishi H, Morisaki H, Liu W, Nakamura H, Morisaki T, Hada T (2005). Recombinant HBsAg inhibits LPS-induced COX-2 expression and IL-18 production by interfering with the NFkappaB pathway in a human monocytic cell line, THP-1. J Hepatol.

[63] Barton GM (2007). Viral recognition by Toll-like receptors. Semin Immunol.

[64] Visvanathan K, Skinner NA, Thompson AJ, Riordan SM, Sozzi V, Edwards R, Rodgers S, Kurtovic J, Chang J, Lewin S, Desmond P, Locarnini S (2007). Regulation of Toll-like receptor-2 expression in chronic hepatitis B by the precore protein. Hepatology.

[65] Isogawa M, Robek MD, Furuichi Y, Chisari FV (2005). Toll-like receptor signaling inhibits hepatitis B virus replication *in vivo*. J Virol.

[66] Chen Z, Cheng Y, Xu Y, Liao J, Zhang X, Hu Y, Zhang Q, Wang J, Zhang Z, Shen F, Yuan Z (2008). Expression profiles and function of Toll-like receptors 2 and 4 in peripheral blood mononuclear cells of chronic hepatitis B patients. Clin Immunol.

[67] Wu J, Meng Z, Jiang M, Pei R, Trippler M, Broering R, Bucchi A, Sowa JP, Dittmer U, Yang D, Roggendorf M, Gerken G, Lu M, Schlaak JF (2009). Hepatitis B virus suppresses toll-like receptor-mediated innate immune responses in murine parenchymal and nonparenchymal liver cells. Hepatology.

[68] Fitzgerald-Bocarsly P, Dai J, Singh S (2008). Plasmacytoid dendritic cells and type I IFN: 50 years of convergent history. Cytokine Growth Factor Rev.

[69] Xie Q, Shen HC, Jia NN, Wang H, Lin LY, An BY, Gui HL, Guo SM, Cai W, Yu H, Guo Q, Bao S (2009). Patients with chronic hepatitis B infection display deficiency of plasmacytoid dendritic cells with reduced expression of TLR9. Microbes Infect.

[70] Li N, Li Q, Qian Z, Zhang Y, Chen M, Shi G (2009). Impaired TLR3/IFN-beta signaling in monocyte-derived dendritic cells from patients with acute-on-chronic hepatitis B liver failure: relevance to the severity of liver damage. Biochem Biophys Res Commun.

[71] Tavakoli S, Schwerin W, Rohwer A, Hoffmann S, Weyer S, Weth R, Meisel H, Diepolder H, Geissler M, Galle PR, Lohr HF, Bocher WO (2004). Phenotype and function of monocyte derived dendritic cells in chronic hepatitis B virus infection. J Gen Virol.

[72] Wang FS, Xing LH, Liu MX, Zhu CL, Liu HG, Wang HF, Lei ZY (2001). Dysfunction of peripheral blood dendritic cells from patients with chronic hepatitis B virus infection. World J Gastroenterol.

